# Effect of Ripasudil on the Change Rates of the Circumpapillary Retinal Nerve Fiber Layer Thickness in Patients With Primary Open-Angle Glaucoma

**DOI:** 10.1155/joph/2962982

**Published:** 2025-01-15

**Authors:** Katsumasa Sakurai, Kenji Suda, Tadamichi Akagi, Hanako Ohashi Ikeda, Takanori Kameda, Masahiro Miyake, Tomoko Hasegawa, Akitaka Tsujikawa

**Affiliations:** ^1^Department of Ophthalmology and Visual Sciences, Kyoto University Graduate School of Medicine, 54 Shogoin-kawahara-cho, Sakyo-ku, Kyoto 606-8507, Japan; ^2^Department of Ophthalmology, Kurashiki Central Hospital, 1-1-1 Miwa, Kurashiki, Okayama 710-8602, Japan; ^3^Division of Ophthalmology and Visual Science, Graduate School of Medical and Dental Sciences, Niigata University, 1-754, Asahimachi-dori, Chuo-ku, Niigata 951-8510, Japan

**Keywords:** glaucoma progression, optical coherence tomography, primary open-angle glaucoma, rho-associated protein kinase inhibitor, ripasudil

## Abstract

**Purpose:** The effect of the Rho-kinase inhibitor ripasudil on the retinal optic nerve fiber layer (RNFL) remains unclear. We aimed to determine this effect in patients with primary open-angle glaucoma (POAG) using optical coherence tomography (OCT) measurements and linear mixed analysis.

**Methods:** This study prospectively included outpatients from a single center with POAG without a history of vitreoretinal or glaucoma surgery from December 2014 to June 2020, in whom the circumpapillary RNFL thickness (cpRNFLT) was measured more than three times before and after ripasudil initiation, without additional medication or surgery during the period. Measurements were performed using OCT in the follow-up mode. The cpRNFLT change rates were compared before and after treatment using linear mixed models with adjustments for intraocular pressure (IOP) changes.

**Results:** Thirty eyes of 20 patients (12 males and eight females) were included. Upon ripasudil prescription, the average cpRNFLT was 60.2 ± 2.1 μm. The average IOP was 15.1 ± 0.5 and 13.5 ± 0.5 mmHg, respectively, before and after treatment initiation, with a difference of −1.6 ± 0.3 mmHg. Analysis of 343 cpRNFLT measurements using linear mixed models revealed that the cpRNFLT change rate was −0.91 ± 0.15 and −0.40 ± 0.14 μm/year, respectively, before and after treatment onset, with an increase of 0.51 ± 0.21 μm/year. After adjusting for IOP changes, the improvement in cpRNFLT change rate was 0.33 ± 0.23 μm/year, albeit not statistically significant.

**Conclusion:** The cpRNFLT change rate significantly increased after ripasudil administration, whereas the contribution of the IOP decline was not significant.

## 1. Introduction

Glaucoma is characterized by intraocular pressure (IOP)-dependent optic nerve damage accompanied by progressive visual field impairment [1, 2]. Because of its high prevalence and irreversible visual field impairment, glaucoma has emerged as a major cause of visual impairment worldwide [3, 4]. The only effective treatment strategy for glaucoma is lowering the IOP, which is achieved through eye drops, laser treatment, or surgery.

Currently, two Rho kinase inhibitors, ripasudil and netarsudil, are available in medical practice; however, ripasudil remains the only approved drug in Japan [5, 6]. Rho kinase inhibitors induce cytoskeleton remodeling, reduce outflow resistance in the Schlemm's canal and trabecular meshwork, and promote the outflow of the aqueous humor from the main outflow tract to lower the IOP [7, 8]. In addition, they may exert vasodilatory and optic nerve-protective effects independent of IOP reduction [9–12].

Two methods are typically used to evaluate the progression of glaucomatous optic neuropathy: detection of visual field progression through static visual field testing and evaluation of the thinning speed of the retinal layer, such as through circumpapillary retinal nerve fiber layer thickness (cpRNFLT) or macular ganglion cell layer-inner plexiform layer measurements using optical coherence tomography (OCT). OCT is notably superior to visual field testing in terms of objectivity and reproducibility, facilitating better quantitative evaluation [13–16].

Ripasudil eye drops have been demonstrated to lower the IOP in some clinical trials [5, 6]. However, the effect of these eye drops on visual field impairment and the structure of the retinal optic nerve fiber layer have not been extensively investigated. Therefore, this study aimed to measure changes in the cpRNFLT thinning rate before and after the instillation of ripasudil eye drops and to examine the factors affecting these changes using a linear mixed model.

## 2. Materials and Methods

### 2.1. Study Design and Participants

Participants were selected from the prospective, single-center Kyoto University Glaucoma Progression Study (UMIN000019854). The study was conducted in accordance with the Declaration of Helsinki and was approved by the Institutional Review Board and Ethics Committee of the Kyoto University Graduate School of Medicine. All participants provided written informed consent before participating in the study.

The participants of this study were the outpatients from the Kyoto University Hospital with primary open-angle glaucoma (POAG) who started using ripasudil eye drops between December 2014 and June 2020. POAG was diagnosed based on the presence of glaucomatous optic neuropathy observed using ophthalmoscopy and OCT, glaucomatous visual field defects corresponding to optic neuropathy, and an open angle on gonioscopy, without other neuro-ophthalmologic or ocular diseases that could cause optic nerve atrophy or visual impairment. IOP was not considered in POAG diagnosis owing to the high prevalence of normal-tension open-angle glaucoma in the Japanese population. Glaucomatous visual field defects were examined using the Humphrey field analyzer (HFA; Carl Zeiss Meditec, CA, US) and defined according to the ocular hypertension treatment study (OHTS) criteria [17]: a cluster of at least three adjacent test points on the pattern deviation probability plot with a *p* value < 5%, with one or more points exhibiting a *p* value < 1%.

Eyes in which cpRNFLT was measured three or more times during the study period without changes in ocular treatment, including eyedrop prescriptions, photocoagulation, or surgery, both before and after the initiation of ripasudil therapy, were included in the study. No restrictions were placed regarding the number of topical medications or IOP levels at baseline. Patients with a history of glaucoma or vitreoretinal surgery, poor OCT image visualization, and whose cpRNFLT could not be accurately measured because of epiretinal membrane (ERM)-induced retinoschisis were excluded.

### 2.2. Data Collection

For each included eye, the following clinical data from the corresponding patient were recorded: laterality, age, sex, best-corrected visual acuity (BCVA; decimal visual acuity measured by Landolt C), refraction (ARK-530A; Nidek, Gamagori, Japan), IOP measurements using Goldmann applanation tonometry, central corneal thickness (CCT) (SP-3000; Tomey, Tokyo, Japan), gonioscopy (including anterior chamber angle), axial length (AL) measurements using ocular biometry (IOL Master; Carl Zeiss Meditec), slit-lamp biomicroscopy, stereoscopic optic disc photography (3-Dx simultaneous stereo disc camera; Nidek, Gamagori, Japan), cpRNFLT measurements using Spectralis HRA + OCT (Heidelberg Engineering, Heidelberg, Germany), mean deviation (MD) values, and foveal sensitivities from the HFA during the study period, type of eye drops used at the start of ripasudil treatment, and length of time without the addition of other treatments before and after initiating ripasudil.

The cpRNFLT was measured using the Spectralis OCT platform, in which the retinal nerve fiber layer was automatically segmented for measurement. Images with obvious segmentation errors due to confusion with the internal plexiform layer, posterior vitreous membrane, or vessel signals were manually corrected by an ophthalmologist (KS). All IOP values were measured using applanation tonometry. The central visual field MD was measured using the 24-2 SITA-Standard method with a Humphrey automatic perimeter. We calculated the average IOPs from three measurements taken before and after ripasudil was initiated, defined as IOPbefore and IOPafter, respectively. The change in IOP (dIOP) was defined as the difference between IOPbefore and IOPafter, using the formula:

(1) $$\begin{array}{c} \text{dIOP}=\text{IOPafter}-\text{IOPbefore}. \end{array}$$

### 2.3. Statistical Analysis

A linear mixed model was employed to evaluate the effects of ripasudil on changes in IOP and the rate of MD change in the HFA. The regression formula included “prepost” as a variable that became zero before the start of ripasudil instillation and one after the start, as follows:

(2) $$\begin{array}{c} {\text{IOP}}_{ij}=\beta_{0}+\beta_{1}\times \text{prepost}+\zeta_{j}+\zeta_{i\left|j\right.}+\varepsilon_{ij},\\ {MD}_{ijt}=\beta_{0}+\beta_{1}\times \text{year}+\beta_{2}\times \text{year}\times \text{prepost}+\zeta_{0j}+\zeta_{0i\left|j\right.}+\zeta_{1i}*\text{year}+\zeta_{1i\left|j\right.}*\text{year}+\varepsilon_{ijt}. \end{array}$$

Changes in the cpRNFLT thinning rate before and after instillation were evaluated using a linear mixed model considering random intercepts and coefficients for both patients and their left and right eyes.

The regression formula was as follows:

(3) $$\begin{array}{c} {\text{cpRNFLT}}_{ijt}=\beta_{0}+\beta_{1}\times \text{year}+\beta_{2}\times \text{year}\times \text{prepost}+\zeta_{0j}+\zeta_{0i\left|j\right.}+\zeta_{1i}*\text{year}+\zeta_{1i\left|j\right.}*\text{year}+\varepsilon_{ijt}, \end{array}$$

where cpRNFLT_*ijt*_ represents individual measurements at visit *t*; *β*_0_, *β*_1_, *β*_2_ represent fixed effects coefficients; *ζ*_0*j*_, *ζ*_1*i*_ represent random patient effects associated with the intercept and time slope; and *ζ*_0*i*|*j*_, *ζ*_1*i*|*j*_ represent random effects associated with the inclusion of both eyes from a single patient.

A linear mixed model was used to evaluate the effects of the changes in IOP caused by treatment with eye drops on the rate of cpRNFLT thinning. The regression formula was as follows:

(4) $$\begin{array}{c} {\text{cpRNFLT}}_{ijt}=\beta_{0}^{\prime }+\beta_{1}^{\prime }\times \text{year}+\beta_{2}^{\prime }\times \text{year}\times \text{prepost}+\beta_{3}^{\prime }\times \text{year}\times \text{dIOP}\times \text{prepost}+\zeta_{0j}+\zeta_{0i\left|j\right.}+\zeta_{1i}*\text{year}+\zeta_{1i\left|j\right.}*\text{year}+\varepsilon_{ijt}. \end{array}$$

The effects of IOPbefore, age, CCT, and AL were evaluated using a linear mixed model. The regression formula was as follows:

(5) $$\begin{array}{c} {\text{cpRNFLT}}_{ijt}=\beta_{0}^{\prime \prime }+\beta_{1}^{\prime \prime }\times \text{year}+\beta_{2}^{\prime \prime }\times \text{year}\times \text{prepost}+\beta_{3}^{\prime \prime }\times \text{year}\times \text{dIOP}\times \text{prepost}+\beta_{4}^{\prime \prime }\times \text{IOPbefore}+\beta_{5}^{\prime \prime }\times \text{Age}+\beta_{6}^{\prime \prime }\times \text{CCT}+\beta_{7}^{\prime \prime }\times \text{AL}+\zeta_{0j}+\zeta_{0i\left|j\right.}+\zeta_{1i}*\text{year}+\zeta_{1i\left|j\right.}*\text{year}+\varepsilon_{ijt}. \end{array}$$

LogcpRNFLT was defined as the logarithm of cpRNFLT, as shown in the following formula [14]:

(6) $$\begin{array}{c} \text{LogcpRNFLT}=10\;\log_{10}\left(\text{cpRNFLT}\right). \end{array}$$

The logarithm of the RNFLT is suggested to have a linear relationship with the total deviation of the visual field sensitivity in the corresponding sector [14]. We used a similar linear model to the aforementioned formulas.

The regression formulas were as follows:

(7) $$\begin{array}{c} {\text{LogcpRNFLT}}_{ijt}=\beta_{0}+\beta_{1}\times \text{year}+\beta_{2}\times \text{year}\times \text{prepost}+\zeta_{0j}+\zeta_{0i\left|j\right.}+\zeta_{1i}*\text{year}+\zeta_{1i\left|j\right.}*\text{year}+\varepsilon_{ijt},\\ \mathrm{Log}{\text{cpRNFLT}}_{ijt}=\beta_{0}^{\prime }+\beta_{1}^{\prime }\times \text{year}+\beta_{2}^{\prime }\times \text{year}\times \text{prepost}+\beta_{3}^{\prime }\times \text{year}\times \text{dIOP}\times \text{prepost}+\zeta_{0j}+\zeta_{0i\left|j\right.}+\zeta_{1i}*\text{year}+\zeta_{1i\left|j\right.}*\text{year}+\varepsilon_{ijt},\\ {\text{LogcpRNFLT}}_{ijt}=\beta_{0}^{\prime \prime }+\beta_{1}^{\prime \prime }\times \text{year}+\beta_{2}^{\prime \prime }\times \text{year}\times \text{prepos}t+\beta_{3}^{\prime \prime }\times \text{year}\times \text{dIOP}\times \text{prepost}+\beta_{4}^{\prime \prime }\times \text{IOPbefore}+\beta_{5}^{\prime \prime }\times \text{Age}+\beta_{6}^{\prime \prime }\times \text{CCT}+\beta_{7}^{\prime \prime }\times \text{AL}+\zeta_{0j}+\zeta_{0i\left|j\right.}+\zeta_{1i}*\text{year}+\zeta_{1i\left|j\right.}*\text{yea}r+\varepsilon_{ijt}. \end{array}$$

Furthermore, we measured cpRNFLT in six sectors (temporal, superotemporal, inferotemporal nasal, superonasal, and inferonasal) and analyzed cpRNFLT and logcpRNFLT values for each sector using the same formulas mentioned above.

All *p* values are presented as 2-sided values. Statistical significance was set at a *p* value < 0.05. All analyses were performed using SAS OnDemand for Academics (SAS Institute Inc., Cary, NC, USA).

## 3. Results

Ripasudil eye drops were administered to 597 patients from December 2014 to June 2020, of whom 129 eyes from 81 patients with POAG underwent three or more cpRNFLT measurements before and after treatment onset. Thirty eyes from 20 patients (12 males and 8 females) had no history of glaucoma or vitreous surgery and had good imaging and accurate cpRNFLT measurements (Table 1). The average age at the start of eye drop treatment was 63.8 ± 11.5 years and the logarithmic minimum angle of resolution (logMAR) visual acuity was −0.01 ± 0.25. An average of 3.5 eye drops were administered, and the average AL of the eyes was 25.4 ± 1.8 mm. The mean follow-up period for cpRNFLT measurements was 2.3 years before and 2.6 years after instillation and the mean number of measurements before and after was 5.6 and 5.9, respectively. The mean IOP was 15.1 mmHg (95% CI: 14.1–16.2 mmHg) before instillation and 13.5 mmHg (95% CI: 12.5–14.6 mmHg) after instillation and a significant decrease was identified using a linear mixed model (−1.6 mmHg; 95% CI: −2.2–−1.0 mmHg; *p* < 0.0001; Table 2). The numbers of HFA (24-2) measurements before and after instillation were 2.4 and 2.1, respectively. Only 10 eyes had undergone multiple measurements both before and after ripasudil treatment, and two eyes had no measurements throughout the study period. The mean MD value at the start of ripasudil treatment was −11.8 dB (95% CI: −14.7–−8.8 dB), with a rate of change of −0.46 dB/year (95% CI: −0.62–−0.29 dB/year, *p* < 0.0001) before instillation and 0.021 dB/year (95% CI: −0.15–0.19 dB/year, *p* = 0.81) after instillation, indicating a significant improvement (*p* = 0.0006; Table 3).

**Figure 1:**
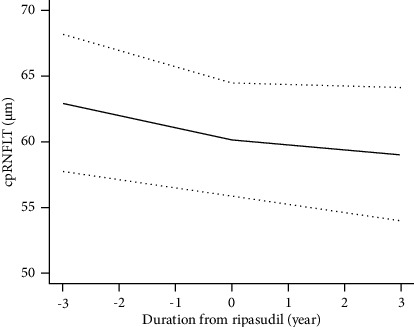
Estimated linear model of longitudinal cpRNFLT change. Estimated cpRNFLT according to the linear model is depicted as the solid line. The thinning rate of cpRNFLT decreased after ripasudil therapy. Dotted lines represent the 95% confidence intervals.

The mean cpRNFLT at the start of ripasudil instillation was 60.2 μm (95% CI: 55.9–64.5 μm). The cpRNFLT thinning rate before starting ripasudil administration was −0.91 μm/year (95% CI: −1.21–−0.61 μm/year, *p* < 0.001) and −0.40 μm/year after starting the eye drops (95% CI: −0.67–−0.12 μm/year, *p* = 0.006). Consequently, the cpRNFLT thinning rate improved by 0.51 μm/year (95% CI: 0.091–0.93 μm/year, *p* = 0.02; Table 4 and Figure 1). The logcpRNFLT thinning rate was −0.061 μm/year (95% CI: −0.085–−0.039 dB/year, *p* < 0.001) before instillation and −0.034 μm/year (95% CI: −0.055–−0.013 dB/year, *p* = 0.002) after ripasudil instillation. Although the thinning rate showed a tendency to improve, no significant difference was observed (*p* = 0.09; Table 5).

Tables 6 and 7 present the analysis results, including the effects of dIOP, IOPbefore, age, CCT, and AL. The effect of dIOP on changes in the cpRNFLT thinning rate was −0.12 μm/year/mmHg (95% CI: −0.25–0.015 μm/year/mmHg, *p* = 0.08), which was not statistically significant. The results of the analyses for logcpRNFLT, sectional cpRNFLT, and logcpRNFLT are shown in Tables 8, 9, 10, and 11.

## 4. Discussion

To assess the effect of ripasudil on lowering IOP in eyes with POAG, Tanihara et al. compared the decrease in IOP 12 and 24 months after the start of eye drop instillation [5, 6]. In the group with an IOP ≤ 15 mmHg before treatment initiation, the IOP decreased by −0.5 ± 0.1 mmHg and −0.6 ± 0.1 mmHg after 12 and 24 months, respectively. In contrast, in the group with an IOP of 15–20 mmHg before the start of instillation, the IOP decreased by −2.1 ± 0.1 and −2.3 ± 0.1 mmHg after 12 and 24 months, respectively. In addition, the decrease in IOP was independent of the number of concomitant glaucoma medications before ripasudil initiation. In the present study, the average IOP before instillation was 15.1 mmHg, exhibiting a −1.6 mmHg decrease after instillation, which is consistent with that of a previous study [5, 6].

Previous studies investigated the rate of cpRNFLT thinning in glaucomatous eyes. Leung et al. [18] analyzed 150 glaucomatous eyes from 90 patients and found that the average cpRNFLT was 70.6 μm, with an average thinning rate of −1.53 μm/year. Similarly, Geng, Wang, and Han [19] examined glaucoma eyes undergoing topical treatment and found that the cpRNFLT thinning rate in the moderate glaucoma group with an average cpRNFLT of 68.01 μm was −0.082 μm/month (−0.98 μm/year), whereas the average cpRNFLT thinning rate in the severe glaucoma group, which had an average cpRNFLT of 48.46 μm, was −0.015 μm/month (−0.18 μm/year). These inconsistencies can be attributed to the floor effect, wherein the thinning rates decrease as glaucoma advances, resulting in a decreased MD score. The results of our study show that the global cpRNFLT at the start of instillation was 60.2 μm, whereas the thinning rate was −0.91 μm/year before instillation and −0.40 μm/year after instillation. Consistent with previous reports, our findings can be interpreted as the natural course of the cpRNFLT thinning rate due to the floor effect. CpRNFLT thinning is also affected by aging; however, Leung et al. reported that the cpRNFLT thinning rate in normal eyes decreased to nearly zero after the cpRNFLT reached below 90 μm [18]. Therefore, the effect of age-related thinning in this analysis was minimal compared with that of glaucomatous thinning, and the changes in thinning rate are assumed to directly reflect changes due to the effects of glaucoma.

Few studies have assessed changes in the cpRNFLT thinning rate in glaucomatous eyes. To the best of our knowledge, no published reports have analyzed changes in the cpRNFLT thinning rate before and after the start of ripasudil instillation. Akagi et al. analyzed changes in the cpRNFLT thinning rate before and after papillary hemorrhage using a linear mixed model and showed that the thinning rate was exacerbated in the sector in which papillary hemorrhage occurred [20]. The present study showed that the cpRNFLT thinning rate improved after the initiation of ripasudil treatment using a linear mixed model. However, our study included more cases of advanced visual field impairment and the observed thinning rate should not be directly compared with that of previous reports. The thinning rate in advanced cases is probably affected by the floor effect than that in nonprogressive cases, as described above, and this rate may decrease regardless of eye drop use. No significant differences were found employing logcpRNFLT, indicating that ripasudil could not prevent the progression of glaucomatous optic neuropathy. However, simply assessing the logarithm results may not be appropriate. Moreover, the sample size may have been too negligible, and it remains unclear whether the ripasudil eye drops had any effect when solely examining changes caused by the floor effect. Therefore, conducting similar analyses with more patients at various disease stages using various eye drops is necessary.

Factors independent of ocular pressure may be involved in the effect of Rho inhibitors on cpRNFLT. Bertrand et al. [9] have shown that Rho inhibitors suppress optic nerve cell apoptosis and promote axonal regeneration, whereas Yamamoto et al. [10] reported that Rho inhibitors suppress apoptosis and the expression of active oxygen species and their synthases in optic nerve cells after axonal injury. From a clinical perspective, Chihara, Dimitrova, and Chihara showed that the parapapillary vascular density was significantly increased with ripasudil compared with brimonidine instillation, although the decrease in tonometry was similar [21]. In the current study, the tonometry decreased significantly before and after the start of ripasudil instillation, accompanied by a significant decrease in the cpRNFLT thinning rate, whereas the effect of the IOP decrease on the rate of decline was not significant. This suggests the possibility of a neuroprotective mechanism that does not depend on an IOP decrease.

Brimonidine inhibits visual field impairment progression better than timolol, despite similar reductions in IOP [22]. Detecting the neuroprotective effects of ripasudil compared with other eye drops may be possible. The linear mixed model used in the present study indicates that of the 0.51 μm/y change in the thinning rate before and after instillation, the IOP decrease accounted for approximately 0.12 μm/y, with a remaining value of approximately 0.39 μm/y. If this value was due to a neuroprotective effect, our results are comparable with those of previous reports, in which citicoline eyedrops reduced glaucoma progression [23]. However, ripasudil was mostly instilled as the third or fourth eyedrop rather than as the first-line treatment, such as that in previous reports. Moreover, our analysis design further differed as it was not a before-and-after comparison of the same patient. In the analysis using the nonlinear model, changes in the thinning rate before and after instillation were not significant. However, further analysis is warranted.

This study had several other limitations. The sample size was relatively minute, hindering the detection of significant differences. In addition, the random effect was not considered in the *β*_2_ term owing to the small sample size. Moreover, it is possible that a group of patients with a relatively good clinical course who did not change treatment regimens for a period of three or more outpatient visits was selected.

In conclusion, ripasudil eye drops not only lowered IOP but also morphologically inhibited the progression of glaucomatous optic neuropathy. Furthermore, the existence of an optic nerve protective effect that does not depend on the lowering of IOP was suggested. However, further analysis is required to elucidate this effect.

## Figures and Tables

**Table 1:** Characteristics of the participants.

Total patients/eyes	20/30
Gender (male/female)	12/8
Age (year)	63.8 ± 11.5
Central corneal thickness (μm)	522 ± 27
Axial length (mm)	25.4 ± 1.8
Best corrected visual acuity (logMAR)	−0.01 ± 0.25
Number of glaucoma medications⁣^∗^	3.5 ± 0.7
Prostaglandin F2*α* agonist	30/30
*β* antagonist	25/30
Carbonic anhydrase inhibitor	26/30
Brimonidine	25/30
History of cataract surgeries	5/30
HFA (24–2)	
Foveal threshold (dB)	32.4 ± 7.3
Medial deviation (dB)	−11.3 ± 7.0
Number of HFA(24–2) examinations	
Before	2.7 ± 2.4
After	2.3 ± 2.1
CpRNFLT at the beginning (μm)	60.2 ± 2.1
LogcpRNFLT at the beginning (dB)	17.7 ± 0.15
OCT follow-up period (year)	
Before	2.3 ± 1.3
After	2.6 ± 1.4
Number of OCT examinations	
Before	5.6 ± 2.2
After	5.9 ± 3.9
Intraocular pressure (mmHg)	
Before	15.1 ± 0.5
After	13.5 ± 0.5
dIOP	−1.6 ± 0.3

*Note:* dIOP: difference in intraocular pressure before and after starting ripasudil, HFA: Humphrey visual field analyzer.

Abbreviations: cpRNFLT, circumpapillary retinal nerve fiber layer thickness; logcpRNFLT: logarithm of cpRNFLT; OCT, optical coherence tomography.

**Table 2:** Intraocular pressure changes between before and after ripasudil therapy.

	B	95% CI	*p*
IOPbefore (mmHg)	15.1 ± 0.5	14.1–16.2	< 0.0001
IOPafter (mmHg)	13.5 ± 0.5	12.5–14.6	< 0.0001
dIOP (mmHg)	−1.6 ± 0.3	−2.2 ∼ −1.0	< 0.0001

*Note:* dIOP: difference of intraocular pressure before and after starting ripasudil, IOPafter: average intraocular pressure for 3 times after starting ripasudil, IOPbefore: average intraocular pressure for 3 times before starting ripasudil.

**Table 3:** Rates of MD changes in HFA (24–2) analyzed by linear mixed model analysis.

	*β*	95% CI	*p*
Average MD (dB)	−11.8 ± 1.4	−14.7 ∼ −8.8	< 0.0001
Rates of MD changes (dB/year)			
Before	−0.46 ± 0.082	−0.62 ∼ −0.29	< 0.0001
After	0.021 ± −0.084	−0.15–0.19	0.81
Change	0.48 ± 0.14	0.21–0.75	0.0006

*Note:* HFA, Humphrey visual field analyzer.

Abbreviation: MD, mean deviation.

**Table 4:** Rates of cpRNFLT change analyzed using linear mixed model analysis.

	*β*	95% CI	*p*
Average cpRNFLT (μm)	60.2 ± 2.1	55.9–64.5	
Rates of cpRNFLT changes (μm/year)			
Before	−0.91 ± 0.15	−1.21 ∼ −0.61	< 0.0001
After	−0.40 ± 0.14	−0.67 ∼ −0.12	0.006
Change	0.51 ± 0.21	0.09–0.93	0.017

Abbreviation: cpRNFLT, circumpapillary retinal nerve fiber layer thickness.

**Table 5:** Rates of logcpRNFLT changes analyzed by linear mixed model analysis.

	*β*	95% CI	*p*
Average logcpRNFLT (dB)	17.7 ± 0.15	17.4–18.0	< 0.0001
Rates of logcpRNFLT changes (dB/year)			
Before	−0.061 ± 0.012	−0.085 ∼ −0.039	< 0.0001
After	−0.034 ± 0.010	−0.055 ∼ −0.013	0.002
Change	0.028 ± 0.017	−0.061–0.005	0.094

Abbreviation: LogcpRNFLT, logarithm of circumpapillary retinal nerve fiber layer thickness.

**Table 6:** Influence of dIOP on rates of cpRNFLT changes.

	*β*	95% CI	*p*
Average cpRNFLT (μm)	60.2 ± 2.1	55.9–64.5	
Rates of cpRNFLT changes (μm/y)			
Before	−0.89 ± 0.15	−1.19 ∼ −0.59	< 0.0001
After	−0.57 ± 0.16	−0.89 ∼ −0.24	0.0012
Change	0.33 ± 0.23	−0.13–0.78	0.16
dIOP effects on the rate of cpRNFLT changes (μm/y/mmhg)	−0.11 ± 0.07	−0.25–0.02	0.097

Abbreviations: cpRNFLT, circumpapillary retinal nerve fiber layer thickness; dIOP, difference in intraocular pressure.

**Table 7:** Multivariate linear mixed model analysis of the factors influencing the rates of cpRNFLT changes.

	*β*	95% CI	*p*
Average cpRNFLT (μm)	60.3 ± 54.9	−52.9–173.4	
Rates of cpRNFLT changes (μm/year)			
Before	−0.89 ± 0.15	−1.19 ∼ −0.59	< 0.0001
After	−0.56 ± 0.16	−0.89 ∼ −0.24	0.0013
Change	0.33 ± 0.23	−0.13–0.79	0.16
dIOP effects on the rate of cpRNFLT changes (μm/year/mmhg)	−0.12 ± 0.07	−0.25–0.02	0.098
IOPbefore (μm/mmHg)	1.40 ± 0.64	0.07–2.72	0.040
Age (μm/year)	0.08 ± 0.19	−0.31–0.46	0.69
Central corneal thickness (μm/μm)	−0.19 ± 0.08	−0.35 ∼ −0.03	0.021
Axial length (μm/mm)	−2.14 ± 1.08	−4.37–0.09	0.060

*Note:* IOPbefore: average intraocular pressure for three times before starting ripasudil.

Abbreviations: cpRNFLT, circumpapillary retinal nerve fiber layer thickness; dIOP, difference of intraocular pressure.

**Table 8:** Linear mixed model analysis of logcpRNFLT and dIOP.

	*β*	95% CI	*p*
Average logcpRNFLT (dB)	17.7 ± 0.15	17.4–18.0	
Rates of logcpRNFLT changes (dB/y)			
Before	−0.060 ± 0.011	−0.082 ∼ −0.037	< 0.0001
After	−0.050 ± 0.012	−0.073 ∼ −0.026	0.0001
Change	−0.010 ± 0.017	−0.024–0.045	0.56
dIOP effects on the rate of logcpRNFLT changes (dB/y/mmhg)	−0.011 ± 0.005	−0.021 ∼ −0.001	0.037

Abbreviations: dIOP, difference of intraocular pressure; logcpRNFLT, logarithm of circumpapillary retinal nerve fiber layer thickness.

**Table 9:** Linear mixed model analysis of logcpRNFLT, dIOP, and other coefficiencies.

	*β*	95% CI	*p*
Average logcpRNFLT (dB)	17.73 ± 3.94	9.62–25.84	< 0.0001
Rates of logcpRNFLT changes (dB/y)			
Before	−0.060 ± 0.011	−0.082 ∼ −0.038	< 0.0001
After	−0.049 ± 0.012	−0.073 ∼ −0.026	0.0002
Change	0.010 ± 0.017	−0.024–0.045	0.55
dIOP effects on the rate of logcpRNFLT changes (dB/year/mmhg)	−0.011 ± 0.005	−0.021 ∼ −0.001	0.037
IOP before (dB/mmHg)	0.104 ± 0.046	0.009–0.199	0.033
Age (dB/y)	0.009 ± 0.014	−0.018–0.037	0.49
Central corneal thickness (dB/μm)	−0.015 ± 0.005	−0.026 ∼ −0.004	0.011
Axial length (dB/mm)	−0.146 ± 0.078	−0.306–0.013	0.071

*Note:* IOPbefore: average intraocular pressure for 3 times before starting ripasudil.

Abbreviations: dIOP, difference of intraocular pressure; logcpRNFLT, logarithm of circumpapillary retinal nerve fiber layer thickness.

**Table 10:** Rates of cpRNFLT changes of 6 sectors analyzed by linear mixed model analysis.

		*β*	95% CI	*p*
T	Average cpRNFLT (μm)	53.4 ± 2.6	48.0–58.7	
	Rates of cpRNFLT changes (μm/y)			
	Before	−0.74 ± 0.21	−1.14 ∼ −0.32	0.0007
	After	−0.54 ± 0.19	−0.92 ∼ −0.17	0.005
	Change	0.18 ± 0.29	−0.39–0.6	0.52

TS	Average cpRNFLT (μm)	75.7 ± 6.4	62.6–88.8	
	Rates of cpRNFLT changes (μm/y)			
	Before	−1.11 ± 0.36	−1.81 ∼ −0.40	0.003
	After	−0.19 ± 0.33	−0.85–0.47	0.57
	Change	0.92 ± 0.46	0.02–1.82	0.044

TI	Average cpRNFLT (μm)	53.0 ± 4.1	44.6–61.5	
	Rates of cpRNFLT changes (μm/y)			
	Before	−1.48 ± 0.39	−2.26 ∼ −0.71	0.0003
	After	−0.44 ± 0.36	−1.16 ∼ −0.28	0.22
	Change	1.04 ± 0.50	0.05–2.03	0.039

N	Average cpRNFLT (μm)	52.7 ± 2.7	47.1–58.2	
	Rates of cpRNFLT changes (μm/y)			
	Before	−0.75 ± 0.25	−1.25 ∼ −0.24	0.0044
	After	−0.12 ± 0.22	−0.58 ∼ −0.34	0.60
	Change	0.63 ± 0.35	−0.07–1.33	0.079

NS	Average cpRNFLT (μm)	72.5 ± 3.9	64.6–80.5	
	Rates of cpRNFLT changes (μm/y)			
	Before	−0.81 ± 0.30	−1.40 ∼ −0.23	0.007
	After	−0.45 ± 0.26	−0.96–0.07	0.086
	Change	0.37 ± 0.42	−0.47–1.21	0.39

NI	Average cpRNFLT (μm)	67.8 ± 4.4	58.7–76.8	
	Rates of cpRNFLT changes (μm/y)			
	Before	−1.07 ± 0.32	−1.71 ∼ −0.42	0.001
	After	−0.65 ± 0.29	−1.25 ∼ −0.06	0.033
	Change	0.42 ± 0.43	−0.43–1.27	0.33

*Note:* TS: superotemporal, TI: inferotemporal, NS: superonasal, NI: inferonasal.

Abbrevitions: T, temporal; N, nasal; cpRNFLT, circumpapillary retinal nerve fiber layer thickness.

**Table 11:** Rates of logcpRNFLT changes of 6 sectors analyzed by linear mixed model analysis.

		*β*	95% CI	*p*
T	Average logcpRNFLT (dB)	17.1 ± 0.24	16.6–17.6	
	Rates of logcpRNFLT changes (dB/y)			
	Before	−0.062 ± 0.019	−0.102 ∼ −0.022	0.002
	After	−0.049 ± 0.018	−0.084 ∼ −0.013	0.008
	Change	0.013 ± 0.028	−0.041–0.068	0.63

TS	Average logcpRNFLT (dB)	18.3 ± 0.38	17.5–19.1	
	Rates of logcpRNFLT changes (dB/y)			
	Before	−0.070 ± 0.028	−0.126 ∼ −0.013	0.016
	After	−0.014 ± 0.026	−0.067–0.039	0.59
	Change	0.056 ± 0.037	−0.017–0.128	0.13

TI	Average logcpRNFLT (dB)	16.9 ± 0.32	16.2–17.5	
	Rates of logcpRNFLT changes (dB/y)			
	Before	−0.129 ± 0.038	−0.204 ∼ −0.055	0.0009
	After	−0.035 ± 0.033	−0.101 ∼ −0.032	0.30
	Change	0.095 ± 0.053	−0.011–0.201	0.078

N	Average logcpRNFLT (dB)	17.0 ± 0.28	16.4–17.6	
	Rates of logcpRNFLT changes (dB/y)			
	Before	−0.058 ± 0.028	−0.115 ∼ −0.001	0.045
	After	−0.037 ± 0.024	−0.086 ∼ −0.011	0.13
	Change	0.021 ± 0.041	−0.061–0.103	0.62

NS	Average logcpRNFLT (dB)	18.1 ± 2.4	17.9–19.0	
	Rates of logcpRNFLT changes (dB/y)			
	Before	−0.048 ± 0.017	−0.083 ∼ −0.013	0.007
	After	−0.027 ± 0.015	−0.057–0.002	0.070
	Change	0.021 ± 0.025	−0.030–0.071	0.42

NI	Average logcpRNFLT (dB)	18.0 ± 0.33	17.3–18.7	
	Rates of logcpRNFLT changes (dB/y)			
	Before	−0.060 ± 0.031	−0.122 ∼ −0.002	0.058
	After	−0.066 ± 0.028	−0.121 ∼ −0.010	0.021
	Change	−0.006 ± 0.044	−0.092–0.080	0.89

*Note:* TS: superotemporal, TI: inferotemporal, NS: superonasal, NI: inferonasal.

Abbreviations: T, temporal; N, nasal; logcpRNFLT, logarithm of circumpapillary retinal nerve fiber layer thickness.

## Data Availability

The data that support the findings of this study are not publicly available because they include information that could compromise the privacy of research participants, but they are available from the corresponding author upon reasonable request.
